# Recent Advances in Electrochemiluminescence Biosensors for Mycotoxin Assay

**DOI:** 10.3390/bios13060653

**Published:** 2023-06-14

**Authors:** Longsheng Jin, Weishuai Liu, Ziying Xiao, Haijian Yang, Huihui Yu, Changxun Dong, Meisheng Wu

**Affiliations:** Department of Chemistry, College of Sciences, Nanjing Agricultural University, 1 Weigang, Nanjing 210095, China

**Keywords:** electrochemiluminescence, mycotoxin, amplification strategy, immunosensor, aptasensor

## Abstract

Rapid and efficient detection of mycotoxins is of great significance in the field of food safety. In this review, several traditional and commercial detection methods are introduced, such as high-performance liquid chromatography (HPLC), liquid chromatography/mass spectrometry (LC/MS), enzyme-linked immunosorbent assay (ELISA), test strips, etc. Electrochemiluminescence (ECL) biosensors have the advantages of high sensitivity and specificity. The use of ECL biosensors for mycotoxins detection has attracted great attention. According to the recognition mechanisms, ECL biosensors are mainly divided into antibody-based, aptamer-based, and molecular imprinting techniques. In this review, we focus on the recent effects towards the designation of diverse ECL biosensors in mycotoxins assay, mainly including their amplification strategies and working mechanism.

## 1. Introduction

With the steady development and expansion of agriculture and animal husbandry, people pay more and more attention to the food safety problems caused by mycotoxin pollution [1,2]. The pollution of mycotoxins in agricultural products and foodstuffs was widespread in the world [3,4]. Agricultural products may be contaminated by mycotoxins during planting, harvesting, processing, transportation, and storage. Furthermore, mycotoxins can also cause the contamination of environmental water through infected crops and aquatic fungi. According to the Food and Agriculture Organization, about 25% of the world’s agricultural products are contaminated every year, causing huge economic losses and seriously endangering human health [5]. Mycotoxins and the secondary metabolite of fungi pose great health risks and threats to animals, plants, and people [6,7,8,9]. These mycotoxins may enter the human body upon the consumption of contaminated foods (milk, eggs, meats, cereals, and fruits), causing serious illness and infections. Additionally, some mycotoxins may lead to liver and kidney damage and are associated with human esophageal cancer [10,11,12].

At present, more than 400 mycotoxins have been found [13]. Among them, the most harmful and widely distributed mycotoxins are aflatoxins, ochratoxins, fumonisins, zearalenone (ZEN), deoxynivalenol (DON), etc. According to the results disclosed by International Agency for Research on Cancer (IARC) [14], Aflatoxin B1 (AFB1) and aflatoxin M1 (AFM1) are classified as group 1 carcinogen and considered the most toxic mycotoxins. Ochratoxin A (OTA) and fumonisins are classified as group 2B possible carcinogens. ZEN and DON are classified as group 3 carcinogens. Many countries have set maximum allowable limits for mycotoxin in food and feed [15,16]. For example, the European Union has set 3 μg/kg for OTA, 750 μg/kg for DON, and 75 μg/kg for ZEA [17]. Many sensitive methods have been established for the analysis of mycotoxins, such as high-performance liquid chromatography-mass spectrometry (HPLC-MS), HPLC, and capillary electrophoresis (CE). However, they cannot meet the demand for convenient and rapid quantification because the samples require complex pretreatment, and the instruments are mostly expensive. Therefore, rapid and efficient detection of mycotoxins is of great significance to ensure food safety.

Enzyme-linked immunosorbent assay (ELISA) kits and lateral flow assay (LFA) test strips are two well-established commercial devices for the rapid detection of mycotoxins. Both are portable, inexpensive, and user-friendly. The results could be read using a simple instrument or the naked eye in several minutes. However, their sensitivity and precision vary by brand, the species of mycotoxins, and the sample matrix [18]. Hu et al. used UHPLC-MS/MS, different commercial ELISA kits, and test strips to detect multiple mycotoxins in cereals [18]. They found that the false negative rate and false positive rate of DON test strips were 30.7% and 26.4%, of FB1 test strips were 37.4% and 31.0%, of ZEN test strips were 25.7% and 8.5%, and of OTA test strips were 0% and 29.8%. For the ELISA kit, the largest deviation ranged from −85.7% to +98.4%, and the RSD was up to 53.6%.

To improve the accuracy and achieve sensitive detection of mycotoxins, sensors have become one of the most effective tools for mycotoxin detection because of their convenient instrumentation, simple operation, and rapid response. Up to now, versatile sensors have been developed for mycotoxin measurement, such as fluorescence [19], colorimetric [20,21], electrochemical [22], chemiluminescence [23], SPR (surface plasmon resonance) [24,25], and Electrochemiluminescence (ECL) [26,27]. Among these techniques, ECL combines the advantages of chemiluminescence and electrochemistry, which have attracted great attention in fabricating the potable and highly sensitive device. Additionally, the optical ECL signal could be captured by a camera or smartphone and analyzed by software or smartphone app. Therefore, in this review, we highlight the research progress of ECL biosensors for mycotoxin detection, including the sensing mechanism, the amplification strategies, and different signal readout strategies.

## 2. Conventional Methods for Determination of Mycotoxin

The most harmful and widely distributed mycotoxins include aflatoxin B1 (AFB1), aflatoxin M1 (AFM1), ochratoxin A (OTA), zearalenone (ZEN), deoxynivalenol (DON), and fumonisin B_1_ (FB_1_). Therefore, many analytical methods have been explored to measure these mycotoxins in food samples, such as high-performance liquid chromatography (HPLC) [28,29,30,31], HPLC-mass spectroscopy (HPLC-MS) [32,33,34], enzyme-linked immunosorbent assay (ELISA) [35,36,37,38], and capillary electrophoresis (CE) [39,40,41,42,43]. Moez’s group employed an aptamer-assisted ultrafiltration purification technique to recognize and separate ochratoxin A (OTA) in green coffee and then use HPLC-fluorescence (FL) detector to realize the quantitative measurement of OTA [44]. Aptamer was first incubated with OTA for 1 h, and then, the mixture was separated by centrifugal ultrafiltration for 30 min. Due to the high binding affinity between aptamer and OTA, the recoveries of OTA in artificially contaminated green tea can reach 97.7%, and the detection limit (LOD) of OTA was 0.05 ng/mL. Luci et al. developed a molecularly imprinted solid phase extraction (MISPEs) method to pre-concentrate and purify targets in pig muscle, liver, and kidney samples [45]. Then, they used HPLC-FL detection technique to achieve the detection of OTA.

HPLC-MS technique combines the effective separation ability of HPLC with the strong component identification ability of mass spectrometry [46]. Kappenberg utilized HPLC-MS method to achieve the simultaneous detection of deoxynivalenol (DON) and zearalenone (ZEA) in a complex soil matrix [47]. The recoveries of DON and ZEA were higher than 80% and 82%, respectively. The detection limits for DON and ZEA were 1 ng and 0.5 ng per gram of soil, respectively. In a work completed by Kudumija et al., a highly sensitive liquid chromatography/tandem mass spectrometry (LC/MS/MS) method was used to analyze the concentration of aflatoxins and OTA in sausages collected from farms [48]. The LODs for AFB1, AFB2, AFG1, AFG3, and OTA were 0.34, 0.40, 0.38, 0.29, and 0.44 μg/kg, respectively.

Enzyme-linked immunosorbent assay (ELISA) is a qualitative and quantitative detection method that utilizes antigen antibody-specific binding for immune response [49]. Liu et al. developed an indirect competitive ELISA for colorimetric detection of OTA in cereals using biotinylated antibody as recognition element and streptavidin-labeled horseradish peroxidase (HRP) as a catalyst [50]. HRP triggered the conversion of tetramethylbenzidine (TMB) to TMB^2+^ which induced the obvious color change of the solution. Compared with traditional ELISA, the sensitivity of this novel ELISA approach has been increased by about 10 times, with an LOD of 0.011 ng/mL.

**Figure 1:**
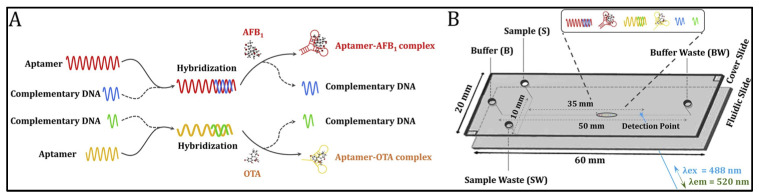
Aptamer-based microchip capillary electrophoresis for trace AFB_1_ and OTA (Reproduced with permission from [51]). (**A**) The measurement procedure of mycotoxin-aptamer complex formation. (**B**) Separation of nucleic acid on MCE-LIF platform.

Capillary electrophoresis (CE) displays outstanding analytical performance in the separation and simultaneous measurement of multiplex targets [51,52]. Liao and co-workers have developed a novel aptamer-based microchip capillary electrophoresis method for the detection of trace AFB_1_ and OTA (Figure 1) [51]. Aptamers were first hybridized with their complementary DNA to form double-stranded DNA. In the presence of the target, the high affinity between the aptamer and target mycotoxin enables the dissociation of dsDNA and the formation of aptamer–mycotoxin complex. These oligonucleotides and aptamer–mycotoxin complexes moved in the capillary at different speeds. Therefore, different types of mycotoxins could be distinguished and detected by capillary electrophoresis after staining with fluorescence dye SYBR gold. The on-chip analysis of aptamer–mycotoxin conjugates could be finished within 3 min with low LODs (0.026 ng/mL for AFB1 and 0.021 ng/mL for OTA).

Although the above analytical methods are sensitive and can realize the simultaneous measurement of multiple targets, HPLC and CE methods mostly have the disadvantages of complicated operation and expensive instruments, and they require well-trained personnel. Thus, an increasing demand is needed to develop convenient and rapid diagnosis techniques in recent years.

## 3. Sensing Strategies in Electrochemiluminescence (ECL) Biosensors for Mycotoxin Analysis

**Figure 2:**
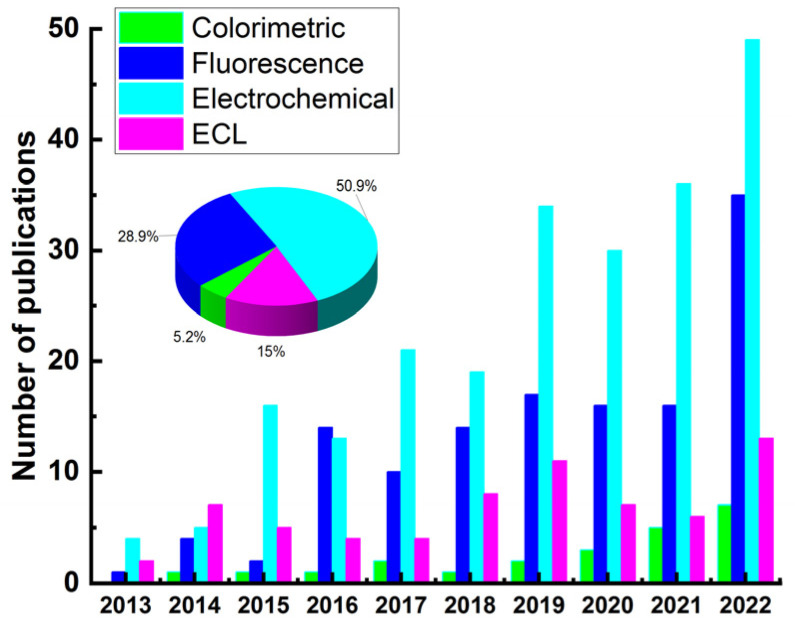
Publications addressing the detection of mycotoxins (aflatoxin, ZEN, OTA, fumonisin, DON) based on sensors (2013–2022).

Figure 2 illustrates the detection techniques reported in the last ten years (2013–2022) for the detection of some typical mycotoxins (aflatoxin, ZEN, OTA, fumonisin, DON) based on sensors. Results were obtained from SciFinder using abstract/keyword “fluorescence”, “colorimetric”, “electrochemical”, and “ECL”, respectively. Although the percentage of total publications for mycotoxin detection using ECL sensor is lower than that of electrochemical and fluorescence sensors, ECL exhibits potential advantages over other optical-based methods because it does not require excitation light source, which not only simplifies the experimental setup but also eliminates the background response of luminescent impurities and scattered light [53]. ECL reactions are potential-controlled, making them controllable and simple operations. Owing to these merits, ECL technology has become one of the most suitable analytical tools for trace target detection in food, environment, disease diagnosis, and so on. According to the recognition elements used, the ECL biosensors could be divided into three categories, including antibodies [54,55,56,57,58,59], aptamers [27,60,61,62,63], and molecular imprinting polymer (MIP) [26,64,65,66].

### 3.1. Antibody-Based Sensing

Immunosensors use antibodies (Ab) as recognition elements, and they exhibit superior sensitivity and selectivity compared to traditional detection methods due to the specific binding of antigens and antibodies. Meanwhile, their operation is relatively simple and they achieve miniaturization, automation, and commercialization easier. Table 1 summarized some ECL-based immunosensors that are used for mycotoxin measurement.

**Figure 3:**
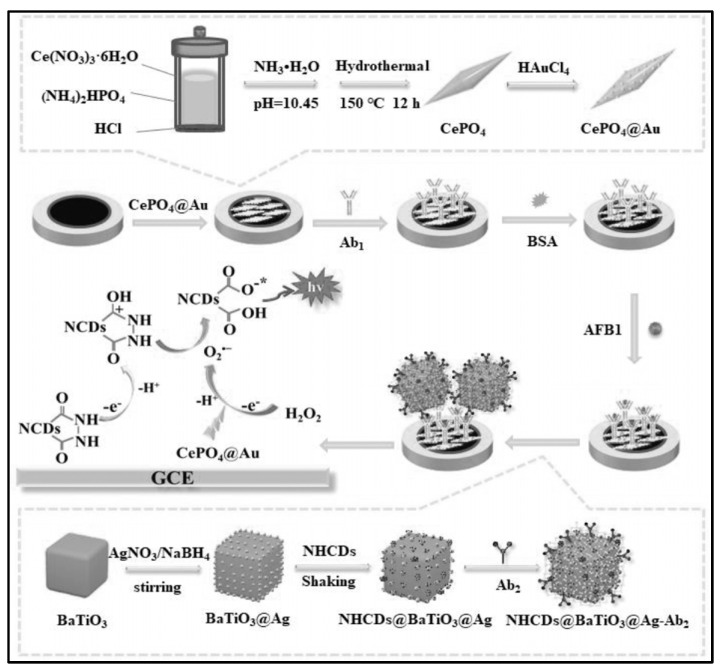
Sandwich−type ECL immunosensor for AFB_1_ detection (Reproduced with permission from [67]).

Li’s group established a sensitive sandwich-type ECL immunosensor for AFB_1_ detection by integrating a synergistic co-reaction acceleration strategy (Figure 3) [67]. CePO_4_@Au was modified on Glassy carbon electrode (GCE) which could provide a large surface area for the decoration of bio-recognition elements (Ab1). Additionally, CePO_4_@Au can catalyze the decomposition of H_2_O_2_ into O_2_^•−^. The produced O_2_^•−^ further reacts with nitrogen-doped hydrazide conjugated carbon dots radicals (NHCDs^•^), thereby greatly amplifying the ECL signal of NHCDs. To further amplify the ECL signal, BaTiO_3_@Ag nanoparticles with excellent conductivity were synthesized which not only provided anchor sites for the immobilization of antibody (Ab2) but also served as an efficient carrier for the modification of NHCDs. The optimal reaction time between antibody and AFB_1_ was 1 h. The constructed biosensor can achieve the accurate detection of AFB_1_ in the range of 0.01 pg/mL–100 ng/mL, and the detection limit was 9.55 fg/mL.

**Figure 4:**
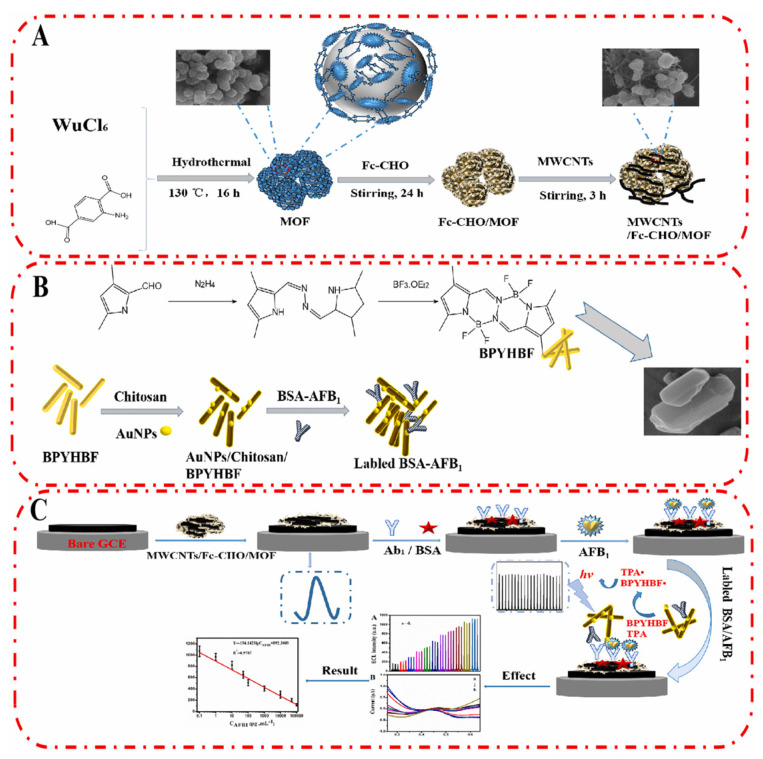
Ratiometric electrochemiluminescence/electrochemical strategy for the ultrasensitive detection of AFB_1_ (Reproduced with permission from [68]). (**A**) Fabrication process of MWCNTs/Fc-MOF. (**B**) Preparation of labeled BSA-AFB1. (**C**) Fabrication process of biosensor.

Fang et al. developed a ratiometric ECL/electrochemical strategy for the ultrasensitive detection of AFB_1_ (Figure 4) [68]. MWCNTs/Fc-CHO/MOF was modified on GCE surface which provides a stable electrochemical signal to calibrate the sensing performance of the immunosensor. Then, a competitive immunosensing interface was constructed by immobilizing the recognition probe (antibody) on MWCNTs/Fc-CHO/MOF surface. In the presence of target (AFB_1_), it would be captured by the antibody with high affinity. When AFB_1_-labeled ECL nanorod was introduced (Au NPs/Chitosan/BPYHBF/BSA-AFB_1_), it competed with AFB_1_ on MWCNTs/Fc-CHO/MOF/Ab1 for the binding sites of Ab1. With the increasing concentration of AFB_1_, the limited amount of Au NPs/Chitosan/BPYHBF/BSA-AFB_1_ would be captured by the modified electrode, leading to a reduced ECL signal. The independent ECL signal and electrochemical signal in response to AFB_1_ could improve the reliability of the biosensor by self-calibration. The linear range of the ratiometric biosensor was from 10 fg/mL to 100 ng/mL, and the detection limit was 5.39 fg/mL. The detection process can be completed in 70 min.

Yang et al. constructed an ECL immunosensor for the detection of OTA in coffee using Au/CaCO_3_ as signal amplification units [54]. Porous hierarchical structure of eggshell was used as template to prepare Au/CaCO_3_. It not only can entrap Au ions and prevent the aggregation of nanoparticles but also provide a large surface for the immobilization of ECL reagent (Ru(bpy)_3_^2+^). OTA biosensor was prepared by modifying Au/CaCO_3_, Ru(bpy)_3_^2+^, and antibody on GCE. In the presence of target mycotoxin, it was captured by the sensing interface, resulting in a remarkable decrease in ECL intensity due to the blocking effect of OTA on interfacial electron transfer. This biosensor can reach the measurement of OTA with a linear range of 10 pg/mL–100 ng/mL and a low detection limit of 5.7 pg/mL.

Kong et al. developed a label-free ECL immunosensor for the determination of AFB_1_ in lotus seed using ZnCdS@ZnS quantum dots (QDs) as ECL emitter [71]. AFB_1_ antibody was immobilized on ZnCdS@ZnS QDs/GCE for specific analysis of AFB_1_. When AFB_1_ was captured by the modified electrode, the resistance was greatly increased, leading to a reduced ECL signal. The applicability of the designed biosensor was tested by monitoring the concentration of AFB_1_ in lotus seed samples. The recoveries of the biosensor were from 99.2% to 101% with RSD < 3.8%, indicating the good performance of the biosensor for mycotoxin detection in real samples. The total detection time was about 30 min.

Wei et al. developed a highly-efficient competitive ECL immunosensor for AFB_1_ measurement [72]. {[Ru(bpy)_3_][Cu_2x_Ni_2(1−x)_(ox)_3_]}_n_ (Cu/Ni/Ru) was used as luminophore. To improve the ECL signal and facilitate the immobilization of recognition probe, Au NPs were labeled on Cu/Ni/Ru. The quantitative determination of AFB_1_ was based on the competitive binding between Au-PEI@SiO_2_-AFB_1_-BSA and free AFB_1_ in solution with AFB_1_ antibody modified on Au-Cu/Ni/Ru. Since PEI could destroy the structure of Cu/Ni/Ru, the combination of PEI@SiO_2_ on Cu/Ni/Ru through the immunoreaction led to the release of Ru(bpy)_3_^2+^ which resulted in the decrease of ECL signal. Under optimal conditions, the biosensor has a linear range from 0.01 to 100 ng/mL with LOD of 39 pg/mL.

Nevertheless, due to the bioactivities of antibodies being susceptible to environmental conditions and the interference of other biological molecules in the immunoreaction process, the cross-reaction is easy to occur in real sample analysis, resulting in false-positive or false-negative results. The detection accuracy and reliability need to be further improved.

### 3.2. Aptamer-Based Biosensor

Unlike antibodies, aptamers are chemically very stable and have simpler storage conditions than antibodies, allowing for longer-term storage. In addition, aptamers have ease of tagging with various molecules. Up to now, versatile amplification strategies based on DNA hybridization techniques could be integrated into aptamer-based biosensors to further improve their sensing performance, such as rolling circle amplification (RCA) [73,74], loop-mediated isothermal amplification [75,76], hybridization chain reaction (HCR) [77,78], DNA walkers [79,80], and so on. Furthermore, nanoparticles with unique physical and chemical properties could be labeled on the terminal of DNA to improve the ECL performance of the biosensor. Therefore, aptamer-based biosensors have attracted great attention in the quantitative detection of mycotoxins. Table 2 summarized some ECL-based aptasensor that are used for mycotoxin measurement.

**Figure 5:**
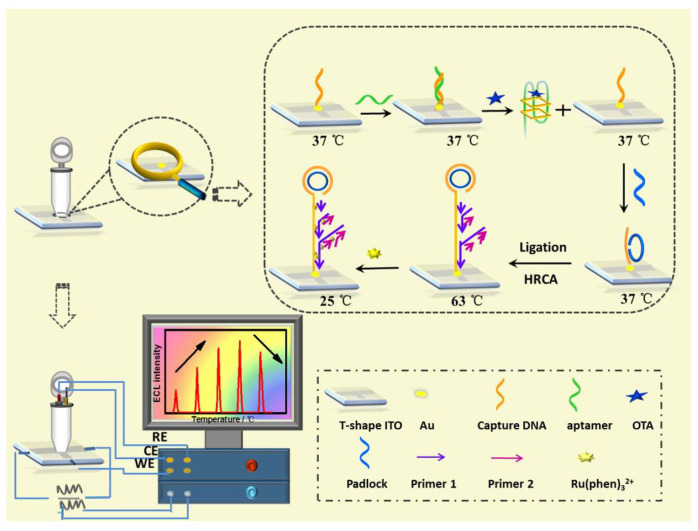
ECL biosensor using hyperbranched RCA strategy for in situ monitoring of OTA (Reproduced with permission from [74]).

Lin et al. developed an ultra-sensitive ECL biosensor using hyperbranched RCA strategy for in situ monitoring of OTA (Figure 5) [74]. CDNA was immobilized on ITO electrode, and then, the temperature of the electrode was raised to 37 °C for the hybridization of aptamer. In the presence of target (OTA), the formation of OTA-aptamer complex caused the release of aptamer from electrode surface, which activated the RCA process. After the extension of DNA sequence and the formation of long dsDNA on electrode surface, a large amount of ECL molecules (Ru(phen)_3_^2+^) could intercalate into the groove of dsDNA, producing an improved ECL signal.

Yuan et al. designed an efficient OTA biosensor using loop-mediated isothermal amplification technique [75]. Aptamer was first reacted with capture DNA on electrode surface to form dsDNA. In the presence of OTA, the remaining aptamers on electrode acted as primer to initiate the loop-mediated isothermal amplification process, causing the intercalation of Ru(phen)_3_^2+^ into dsDNA. The accumulation of plenty of ECL molecules on electrode surface greatly amplifies the ECL signal and improves the sensitivity of the biosensor. As a result, an ultra-low LOD of 10 fM and a wide linear range of 0.00005 nM~100 nM was obtained for OTA detection.

**Figure 6:**
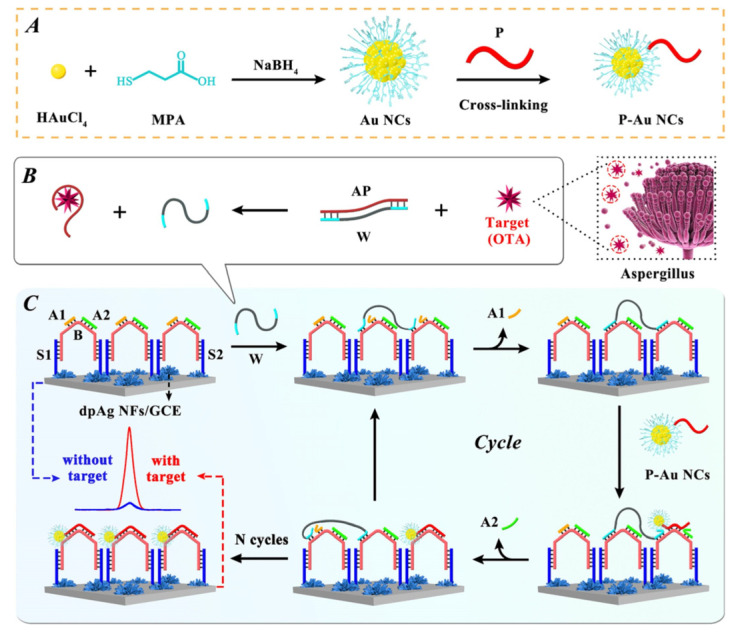
Non-enzymatic ECL biosensor for the detection of OTA based on the efficient amplification strategy of DNA walker (Reproduced with permission from [80]). (**A**) Preparation of monodispersed MPA-Au NCs. (**B**) The target transformation process. (**C**) Schematic diagram of proposed ECL biosensor for OTA detection.

Yuan et al. developed a non-enzymatic ECL biosensor for the detection of OTA based on the efficient amplification strategy of DNA walker (Figure 6) [80]. Au nanoclusters (NCs) were used as ECL signal probes and labeled on the terminal of DNA P. The hybridization of DNA B with DNA P on Au NCs forced the release of bipedal DNA walker and the autonomous movement of DNA walker along the DNA distributed on the electrode surface, thus achieving the ultra-sensitive detection of OTA. Wei et al. proposed a simple and sensitive ECL aptasensor for the determination of OTA based on a nicking endonuclease-powered DNA walking machine [89]. The presence of target activated the nicking endonuclease which powered the walking of DNA, forcing the release of Cy5 from electrode surface. As a result, the ECL energy transfer from ECL donor (CdS QDs) to ECL acceptor (Cy5) was suppressed. The aptasensor can detect OTA in a range from 0.05 nM to 5 nM with a detection limit of 0.012 nM.

**Figure 7:**
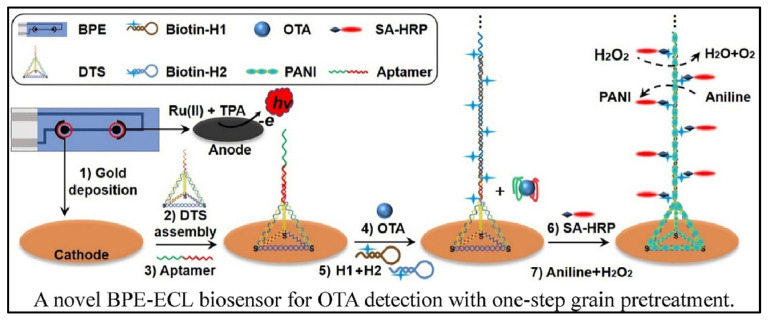
Bipolar electrode-ECL device for OTA detection based on HCR amplification (Reproduced with permission from [78]).

Cao et al. designed a bipolar electrode (BPE)-ECL device for OTA detection with one-step grain pretreatment based on DNA tetrahedron-structured aptamer and the amplification effect of HCR (Figure 7) [78]. DTS structure on electrode surface can not only provide anchor sites for the HCR but also optimize the trigger DNA density. In the presence of a target, the aptamer was separated from electrode surface which trigged the extension of DNA sequence with the help of two types of hairpin-structured DNA. Owing to the biotin tag on the terminal of hairpin DNA, the extension of DNA sequence provided a large number of reaction sites for the combination of streptavidin-HRP (SA-HRP). HRP catalyzed the polymerization of aniline to form polyaniline (PANI), resulting in the significant change of ECL intensity and luminescence voltage of the anode of BPE. Although it takes about 8 h to finish the detection of OTA, the biosensor can realize the detection of OTA down to 3 pg/mL. In addition, the accuracy of the biosensor was similar with HPLC analysis.

Wang et al. developed a nuclease-driven signal amplification strategy for label-free measurement of AFB_1_ [90]. Ru(bpy)_3_^2+^-Probe (Ru-Probe) was adsorbed on magnetic graphene oxide (M-GO). The aptamer was reacted with a catalyst probe to form dsDNA. After the introduction of the target, the formation of aptamer:target complex led to the release of the catalyst probe. The catalyst probe further hybridized with Ru-Probe and initiated the cycling cleavage of Ru-Probe by exonuclease III. Due to the nuclease-driven signal amplification process, the detection limit of this biosensor can reach 20 fM.

Apart from the application of DNA amplification technologies, other sensing mechanisms were also proposed, such as label-free, small molecules and nanoparticle amplification strategies. Kong et al. developed a label-free ECL aptasensor for OTA detection in Lily and Rhubarb samples [91]. The biosensor consisted of chitosan/CdSe@CdS quantum dots (QDs)-modified Au electrode. OTA aptamers were immobilized through the coupling of glutaraldehyde. In the presence of OTA, the formation of OTA–aptamer complex on electrode surface inhibited the ECL intensity greatly. Ding et al. developed a regenerable biosensor based on the quenching effect of Ferrocene (Fc) on the ECL of Graphitic carbon nitride (g-CN) for the detection of AFB_1_ [60]. The Fc-labeled aptamer was modified on g-CN/GCE surface. After reacting with AFB_1_, the conformation changes of the aptamer enhanced the quenching efficiency of Fc on the ECL signal of g-CN. Meanwhile, the combined AFB_1_ on Fc-aptamer/g-CN/GCE could also react with the high energy state of g-CN, causing the further decrement of ECL signal. Due to the dual-quenching effect, the biosensor enables the detection of AFB_1_ in the range from 0.005 ng/mL to 10 ng/mL. The attractive property of the biosensor was that it could be regenerated by increasing the temperature to 40 °C. Results indicated that within 5 times regeneration, the deviation rate was less than 5%.

In a recent work by our group, we fabricated a portable ECL device using MWNTs-PDMS flexible fiber as bipolar electrode and the catalytic effect of Ag NPs to achieve the sensitive detection of FB_1_ [27]. By adjusting the ratio of MWNTs and PDMS, the conductivity and the stiffness of the fiber could be easily controlled. The fiber electrode could be cut into desired lengths. A MWNTs-PDMS BPE electrode array consisting of 6 fibers was fabricated, and Au NPs were deposited on one pole of it through bipolar deposition. The excellent conductivity and large surface area of Au NPs caused an 89-fold enhancement of ECL signal compared to that of bare BPE. When Ag NPs were decorated on electrode surface through the thiol group on the aptamer, the ECL signal could be further improved to 13.8-fold. The excellent amplification effect of Au NPs and Ag NPs enables a wide range of the biosensor for sensing FB_1_.

**Figure 8:**
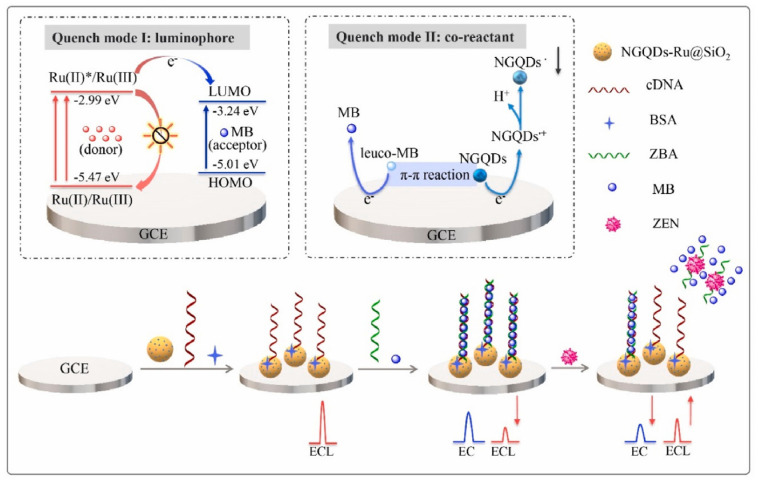
ECL−electrochemical ratiometric aptasensor based on the dual quenching effects of MB (Reproduced with permission from [83]).

To reduce the false-positive response, ratiometric biosensors have been developed recently [77,92,93]. The ratio between two different signals can provide an intrinsic calibration function for target detection, which improves the accuracy of the biosensor. If these two signals exhibited opposite alternation in response to target, the sensitivity could be greatly improved. Therefore, using the ratiometric signal as the output can also broaden the linear range and reduce the detection limit of the biosensor. You et al. prepared an ECL-electrochemical ratiometric aptasensor based on the dual quenching effects of methylene blue (MB) for the determination of ZEN (Figure 8) [83]. Ru@SiO_2_ (Ru(bpy)_3_^2+^-doped silica nanoparticles) was used as ECL donor. NGQDs (nitrogen-doped graphene quantum dots) were modified on Ru@SiO_2_ surface to act as a self-enhanced co-reactant. MB was used as ECL quencher due to the ECL energy transfer between Ru@SiO_2_ to MB. Furthermore, it can interact with NGQDs via π-π conjugation and inhibit the generation of NGQDs’s intermediates, leading to the further inhibition of ECL signal. The dual-quenching effects of MB were realized by the adsorption of large amounts of MB molecules on the dsDNA structure on NGQDs-Ru@SiO_2_/GCE. After the introduction of target, it induced the deformation of dsDNA, causing the liberation of MB from electrode surface and the recovery of ECL signal. Based on the ratio of the “turn-on” ECL signal and “turn-off” electrochemical signal of MB, the ratiometric biosensor exhibited a low detection limit of 0.85 fg mL^− 1^. Jie et al. developed a dual-signal (ECL and electrochemical) biosensor for Dam methylase (MTase) and AFB_1_ assay based on a multifunctional DNA nanotube [94]. The ECL signal and electrochemical signal were generated by the MB and Ru(phen)_3_^2+^ molecules in dsDNA on DNA nanotube, respectively. A specific Dam MTase sensing interface was fabricated by immobilizing hairpin DNA on gold electrode. The presence of target Dam MTase induced the methylation of the hairpin-structured DNA, which was then cleaved into single-strand DNA in the presence of endonuclease DpnI. When ECL signal probes (Ru(phen)_3_^2+^-DNANT) or electrochemical signal probes (MB-DNANT) were introduced, they could be captured by the single-strand DNA on the electrode. After the introduction of AFB_1_, both the ECL and electrochemical signals were turned off.

Although the aptamer-based biosensor has the advantages of high sensitivity, high selectivity, and low batch-to-batch variation, it also has some inherent disadvantages: (1) the electrode assembly process is laborious and time-consuming, and a series of optimization of the assembly conditions are needed to be performed; (2) the binding of biometric elements at the sensing interface will weaken over time, resulting in insufficient long-term stability of the modified electrode.

### 3.3. Molecular Imprinting

In addition to the antibody and aptamer recognition methods described above, the molecular imprinting technique-based recognition methods have attracted considerable attention due to their unique advantages, such as simplicity, rapidity, reusability, and high selectivity [95,96,97]. Usually, the molecular imprinting polymer (MIP) recognition process involves (1) a homogeneous pre-polymerization complex prepared by incubating template molecules with monomers; (2) polymerization of the monomers under photo-/thermal conditions to encapsulate template molecules inside the polymeric matrix; and (3) the removal of trapped template molecules with elution solvents, leaving specific cavities with the same shape of templates. The MIPs containing specific cavities have excellent selectivity for targets with similar structures and shapes of templates. Due to the specific recognition mechanism, MIP strategies have been employed for label-free detection of mycotoxins [66,98,99,100].

Zhang et al. developed a novel molecularly imprinted ECL sensor using Ru@SiO_2_ NPs as ECL emitters [26]. To amplify the ECL signal, a large number of ECL molecules (Ru(bpy)_3_^2+^) were encapsulated in SiO_2_ NPs. MIP materials were prepared using FB_1_ as template molecules. After the formation of MIP materials, FB_1_ molecules were eluted, and the porous MIP materials were used as the template for the recognition of FB_1_ in samples. To improve the sensitivity of the biosensor, Au NPs were modified on electrode surface to enhance the ECL intensity due to its localized surface plasmon resonance (LSPR) and excellent electrochemical properties. As a result, the MIP-based ECL biosensor exhibited high sensitivity and selectivity for FB_1_ detection with a low detection limit of 0.35 pg/mL.

Zhang and co-workers developed an ECL resonance energy transfer system for OTA detection using MIP as a recognition element [65]. Ru(bpy)_3_^2+^-doped silica NPs were modified on the electrode and acted as ECL donors. CdTe QDs were used as the ECL acceptor which could amplify the ECL intensity. The cavities in MIPs can not only selectively recognize the target but also work as a tunnel to facilitate the transfer of co-reactant to electrode surface to activate the ECL signal. When the target was introduced, it blocked the tunnels, leading to the dual-quenching effect of ECL signal: the inhibited energy transfer from Ru(bpy)_3_^2+^ to CdTe QDs and the restricted accessibility of co-reactant from solution to electrode surface. Owing to the dual-quenching effect, the biosensor enables the detection of OTA with a wide range from 1.0 × 10^−5^ to 11.13 ng/mL and a lower detection limit of 3.0 fg/mL.

**Figure 9:**
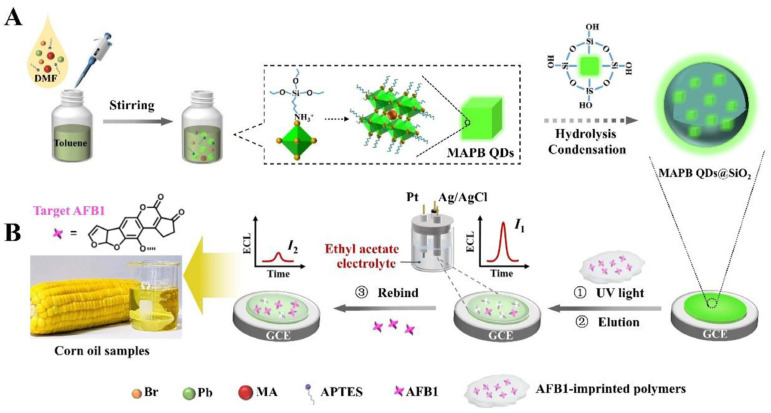
The preparation (**A**) and working (**B**) process of MAPB QDs@SiO_2_-based MIP-ECL sensors for AFB_1_ detection (Reproduced with permission from [100]).

Chen et al. developed a silica-encapsulated CH_3_NH_3_PbBr_3_ quantum dots (MAPB QDs)@SiO_2_-based MIP-ECL sensor for AFB_1_ detection in corn oil samples (Figure 9) [100]. The encapsulation of luminophore in SiO_2_ particles greatly improved the ECL signal. After immobilizing MAPB QDs@SiO_2_ and MIP film on electrode surface and removing the template molecules, the biosensor can realize sensitive and label-free detection of AFB_1_. By exploring the nanoparticle-based amplification strategy and the MIP-based recognition technique, the prepared biosensor can detect AFB_1_ with an ultra-low detection limit of 8.5 fg/mL. The biosensor was then used for corn oil sample analysis using the standard addition method. The recoveries were from 101.7% to 106.7%, indicating the good performance of the biosensor in real sample detection. Additionally, the biosensor exhibited excellent long-term storage stability.

Metal–organic frameworks (MOFs) were also used to construct MIPs because they possess adjustable three-dimensional structures and are easy to be functionalized, making them an attractive material for the encapsulation of ECL molecules and nanoparticles. Chen and co-workers embedded CH_3_NH_3_PbBr_3_ QDs in ZIF-8 MOFs and developed an ultra-stable imprinted polymer for FAB_1_ detection [89]. Results revealed that the encapsulation of QDs in ZIF-8 MOFs could greatly improve ECL stability. This is because the pore structure of ZIF-8 MOFs can inhibit the external perturbation from polar water and could avoid the dissolution of QDs effectively. Then, a specific imprinted polymer membrane was constructed on QDs@ZIF-8/GCE for target detection. Due to the improved stability of the ECL indicator and the improved sensitivity, the biosensor demonstrated a wide linear range from 11.55 fg/mL to 20 ng/mL for FB_1_ detection and a low detection limit of 3.5 fg/mL. The sensing performance of the biosensor was also compared with HPLC method. Results clearly showed that the MIP-based biosensor has a higher sensitivity than that of HPLC.

Compared with natural recognition elements such as antibodies and aptamers, MIPs have the advantages of mild storage conditions, long-term preservation, and lower cost. In addition, MIPs are more resistant to extreme pH, variable temperature, and high ionic strength but have the disadvantages of average sensitivity, low recognition ability for macromolecular targets, and relatively complicated preparation of MIPs.

### 3.4. Visual ECL Analysis

**Figure 10:**
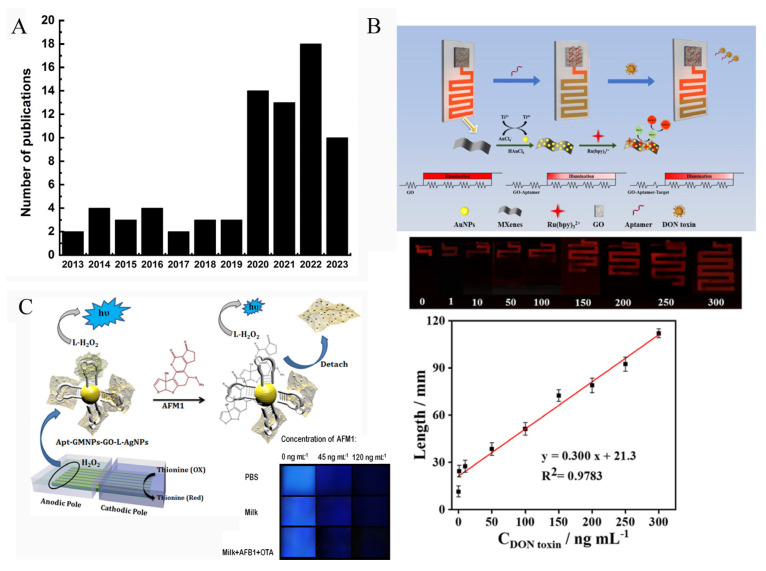
(**A**) Number of publications published each year over the last 10 years on the topic of visual ECL devices. (**B**) Schematic representation of portable ECL sensor for detection of DON (Reproduced with permission from [103]). (**C**) Amplified visual ECL detection of AFM1 based on a smartphone (Reproduced with permission from [88]).

As a powerful analytical method, a very important advantage of ECL is that the strong ECL emission and the obvious color change of ECL signal could be observed by the naked eye or visual inspection by CCD (charge-coupled device) camera or a portable detector, such as a smartphone. Then, the ECL information of the images is analyzed by software or a smartphone-based app. When an electrode array was used to fabricate the biosensor, the ECL signals emitted from each electrode could be collected and analyzed simultaneously. These outstanding features of ECL technique hold great promise in point-of-care diagnosis [101,102]. Figure 10A displays the number of publications related to visual ECL devices. It clearly demonstrates that the ECL imaging technique has received increased attention.

Wang’s group developed a visual ECL biosensor chip (2 × 4 cm^2^) that can convert the concentration of a target into a distance signal (Figure 10B) [103]. The chip device contained a detection region and a distance readout channel region. Graphene oxide (GO) and AuNPs@Ti_3_C_2_ nanocomposites were modified on these two specific regions, respectively. When the recognition reaction between target (OTA) and aptamer was imitated, the released aptamer would be released from GO surface, which changed the potential of the channel region, resulting in the change of the lighting length of the channel. By measuring the length of the channel that can emit ECL signal, the OTA concentration could be easily obtained.

Bagheri et al. developed a sensitive smartphone-based ECL biosensor based on luminol-functionalized, silver nanoparticle-decorated graphene oxide for AFM1 detection (Figure 10C) [88]. They prepared an array of seven parallel gold bipolar electrodes (25 × 25 mm) on a gold CD (25.0 × 30.0 mm). For visual analysis, a glass cell (8.0 cm length and 2.5 cm width) containing cathodic and anodic channels was designed. Then, the anodic pole of the electrode was modified with Apt-GMNP-GO-L-AgNPs which could amplify the ECL signal of luminol/H_2_O_2_ system. In the presence of the target (AFM1), the combination of aptamers with AFM1 caused the detachment of GO-L-AgNPs from electrode surface, leading to a reduced ECL signal. The remarkable ECL change could be recorded by both photomultiplier tube (PMT) and smartphone. Figure 10C shows the ECL signal recorded by smartphone, and the brightness value of the image was analyzed using ImageJ software. The gray value of the ECL image demonstrated a good linear relationship with AFM1 concentration from 10 to 200 ng/mL.

Liu et al. proposed a multicolor BPE-ECL sensor for the visual detection of OTA based on the ECL color change of Ru(bpy)_3_^2+^ and Ir(ppy)_3_^2+^ at the anode of BPE [104]. Fc-aptamer was modified on the cathode of BPE through base complementation. Then, it was electrochemically oxidized to Fc^+^ which could enhance the ECL signal of both Ru(bpy)_3_^2+^ and Ir(ppy)_3_^2+^ at the anode. The presence of OTA forced the release of Fc from the electrode, causing the color change from dark orange to forest green.

Overall, all these ECL biosensors show broad linear range and low detection limit for mycotoxin detection. Because of the operational simplicity and the smartphone-based readout system, the visual ECL biosensor holds great promise in commercial POC use. In addition, versatile amplification strategies could be employed to improve the sensitivity of the biosensor.

## 4. Conclusions and Perspectives

This review introduced the most recent progress toward mycotoxin diagnosis. Conventional technologies, such as HPLC, HPLC-MS, and CE, have high sensitivity and specificity for mycotoxin detection. However, they rely on expensive analytical instruments and require training personnel. To achieve rapid and convenient measurement, ELISA kits and test strips are developed and commercially available. They can fulfill point-of-care detection without special detectors. Due to their simplicity, low cost, and fast response time, these two approaches have been widely used in the rapid analysis of mycotoxins outside of the laboratory. However, their precision and sensitivity vary by brand, which increases the probability of false positive and false negative results. To make the result more convincing and improve the sensitivity, versatile sensors have been developed. Among them, ECL biosensors have attracted promising attention in the field of mycotoxin determination due to their high sensitivity, selectivity, and reproducibility.

ECL biosensors typically consist of an electrode integrated with a recognition element, signal amplification unit, and optical readout system. This review focused on the progress of antibody-based, aptamer-based, and MIP-based ECL sensors. Both antibody and aptamer have high binding affinity with target. The commonly used amplification strategy in antibody-based biosensors relies on the introduction of nanoparticles, while for aptamer-based biosensors, versatile amplification strategies could be employed, such as DNA-based amplification strategies and nanoparticles. For MIP-based sensors, it eliminates the utilization of high-cost biorecognition probe and could be regenerated.

Despite significant progress having been achieved, there is still a desire for developing novel miniaturized ECL biosensors based on new amplification principles and highly sensitive readout systems. With the fast development of nanotechnology and smart devices, advanced ECL biosensors having a high throughput readout capability would become one of the important directions for fabricating portable devices. By designing an electrode array with independent sensing elements, the optical detector can acquire multiple distinct signals simultaneously in a single measurement.

In addition, the challenges for accurately detecting mycotoxin lie in the complexity of the sample matrix, which may produce a false-positive signal. To improve the accuracy and the reproducibility of the biosensor, the development of versatile and sensitive ratiometric strategies that can provide built-in self-calibration functions would become the hotspot in this research field.

Although antibodies, aptamers, and MIP are commonly used as efficient recognition probes for mycotoxins, this review has revealed that very few MIP-based ECL biosensors are developed for mycotoxin measurement. Moreover, little attention has been paid to the regeneration and reusability of MIP-based biosensors. Therefore, great effort should be paid to the reusability of MIP-based biosensors in multicycle analyses. Future studies on novel ECL biosensors should try to employ dual-recognition strategies based on the combination of MIPs and biorecognition probes, which could improve the sensitivity, binding affinity, and selectivity of the biosensor. Importantly, it could improve the stability of the biosensor under harsh environmental conditions.

Additionally, to realize the commercialization of ECL biosensors, the development of biosensors integrated with multiple functions such as temperature control, sample pretreatment, and self-calibration is of great importance.

## Figures and Tables

**Table 1:** Summary of various antibody-based ECL biosensors for mycotoxins detection.

Sensing Mechanism	Target	Linear Range (ng/mL)	LOD (fg/mL)	Real Sample	Ref.
Potential-resolved	AFB_1_	5 × 10^−5^ to 100	16.44	Walnut	[58]
Sandwich-type ECL immunosensor	AFB_1_	1 × 10^−5^ to 100	9.55	Corn, rice, and wheat	[67]
Competitive immunosensor (ratiometric ECL/electrochemistry)	AFB_1_	1 × 10^−5^ to 100	5.39	Walnut	[68]
Self-enhanced electrochemiluminescent ratiometric immunosensor	ZEN	1 × 10^−4^ to 10	33	Corn hazelnut	[69]
Peptide-based competitive immunoassays	ZEN	1 × 10^−5^ to 0.1	3.3	Coconut milk	[70]
Resistance-induced ECL change	OTA	0.01 to 100	5.7 × 10^3^	Coffee	[41]
Resistance-induced ECL change	AFB_1_	0.05 to 100	1 × 10^4^	Lotus seed	[71]
Competitive immunosensor	AFB_1_	0.01 to 100	39 × 10^3^	Fresh milk	[72]

**Table 2:** Summary of various aptamer-based ECL biosensors for mycotoxins detection.

Amplification Mechanism	Target	Linear Range	LOD (fg/mL)	Real Sample	Ref.
RCA	OTA	0.075 to 10 pg/mL	8 fg/mL	Red wine	[74]
HCR	OTA	0.01 ng/mL to 5 ng/mL; 5 ng/mL to 100 ng/mL	3 pg/mL	Rice, wheat, corn, sorghum, barley, buckwheat	[78]
HCR and Dual-signal amplification strategy	OTA	12.40 pM to 6.19 nM	5.6 pM	Corn	[77]
DNA walker	AFB_1_	1.0 fg/mL to 10 ng/mL	0.58 fg/mL	Corn, peanut	[79]
DNA walker	OTA	10 fg/mL to 100 ng/mL	3.19 fg/mL	Corn oil, rapeseed oil, sesame oil	[80]
Loop-mediated isothermal amplification	OTA	0.00005 nM to 100 nM	10 fM	Red wine	[75]
ECL energy transfer	AFB_1_	5.0 pM to 10 nM	0.12 pM	Peanut, maize, wheat	[81]
ECL energy transfer	OTA	0.0005 to 50 ng/mL	0.17 pg/mL	Corn	[82]
Dual-signal amplification strategy	ZEN	0.001 to 200 μg/kg	9.75 × 10^−5^ μg/kg	Maize	[62]
Dual-quenching effects	ZEN	1.0 × 10^−6^ to 50 ng/mL	0.85 fg/mL	Maize	[83]
Dual signal amplification of GQDs and AuNRs	AFB_1_	0.01 to 100 ng/mL	3.75 pg/mL	Peanut, maize, wheat	[84]
Exonuclease-assisted target recycling amplification	OTA	0.01 to 1.0 ng/mL	2 pg/mL	Corn	[85]
Horseradish peroxidase	AFB_1_	0.1 to 100 ng/mL	0.033 ng/mL	Rice, wheat, corn, sorghum, barley, buckwheat	[86]
Self-enhanced NPs (NGQDs- Ru@SiO_2_)	ZEN	10 fg/mL to 10 ng/mL	1 fg/ mL	Corn flour	[87]
Apt-GMNPs-GO-L-AgNPs	AFM_1_	10 to 200 ng/mL	0.05 ng/mL	Milk	[88]

## Data Availability

No new data were created or analyzed in this study. Data sharing is not applicable to this article.
